# Increased *cis*-to-*trans* urocanic acid ratio in the skin of chronic spontaneous urticaria patients

**DOI:** 10.1038/s41598-017-01487-9

**Published:** 2017-05-02

**Authors:** Duy Le Pham, Kyung-Min Lim, Kyung-Mi Joo, Hae-Sim Park, Donald Y. M. Leung, Young-Min Ye

**Affiliations:** 1 Department of Allergy and Clinical Immunology, Ajou University School of Medicine, Suwon, Republic of Korea; 2 College of Pharmacy, Ewha Womans University, Seoul, Republic of Korea; 3 AmorePacific R&D Center, Yongin, Republic of Korea; 4 Department of Pediatrics, National Jewish Health, Denver, Colorado USA

## Abstract

Increased filaggrin expression was found to be correlated with severity scores in chronic spontaneous urticaria (CSU); however, the role of filaggrin breakdown products (FBPs) in CSU has not been studied. We collected stratum corneum (SC) specimens from the volar forearms of 10 CSU patients, 10 AD patients, and 10 healthy normal controls (NCs) and measured contents of FBPs (pyrrolidone carboxylic acid [PCA] and urocanic acid [UCA]) using UPLC-MS/MS, transepidermal water loss (TEWL) and epidermal pH. Compared to NCs, *cis*-UCA level was increased in CSU lesions (*P* < 0.05) and decreased in AD lesions (*P* < 0.01). The *cis-*to-*trans-*UCA ratio in SC specimens from CSU patients was significantly greater than those from AD and NC subjects. AD lesions had lower FBP and PCA contents compared to NC skin (both *P* < 0.001), and higher TEWL and pH compared to CSU lesions. Moreover, *cis*-UCA, but not *trans*-UCA, enhan*c*ed the IgE-mediated basophil activation, as well as IgE- and calcium-mediated degranulation of LAD-2 cells, in a dose-dependent manner. These findings suggest that increased *cis*-to-*trans* UCA ratio in the epidermis is a distinct feature of CSU, which could enhance mast cell degranulation. Modulation of *cis*-UCA may be a potential target for skin diseases associated with IgE-mediated mast cell degranulation.

## Introduction

Chronic spontaneous urticaria (CSU) is defined as the repeated occurrence of transient (less than 24 hours) wheals and/or angioedema for at least 6 weeks without an eliciting cause^1^. Recent guidelines recommend identification and avoidance of the underlying causes of chronic urticaria (CU) as the main goal of treatment^1^. However, in the majority of patients, finding and eliminating the causes of CSU are difficult. Although the pathogenic mechanism of CSU has yet to be completely elucidated, the main pathophysiology involves the activation of mast cells and basophils. The release of mast cell mediators induces inflammation and the activation of other inflammatory cells, such as eosinophils, neutrophils, and T cells^2^.

Chronic urticaria is a heterogeneous disease with various phenotypes. Each subset may be associated with various triggers and different pathways of immune activation. In addition to the 45% of patients with autoimmune features, 55% of CSU patients have unidentified specific inducers for mast cell degranulation and associated inflammation^3^. Accordingly, standard treatment with antihistamines is often ineffective, as around 40% of CU patients show poor response to antihistamines^4–6^. Moreover, therapeutic responses to anti-IgE antibody treatment also differ among patients^7^. Thus, the development of suitable biomarkers may help identify patients who would benefit from early application of precision medicine approaches.

Previously, we documented upregulated expression of filaggrin at both mRNA and protein levels in the skin of CSU patients, compared to skin from atopic dermatitis (AD) patients^8^. AD is associated with decreased filaggrin expression, resulting from abnormalities in the terminal differentiation of the epidermis and filaggrin gene mutation, leading to enhanced allergen penetration, systemic IgE sensitisation, and recurrent microbial infection^9^. Some clinical features (e.g., itching) and histological characteristics (e.g., infiltration of T lymphocytes, eosinophils, and mast cells into skin lesions) are shared between CSU and AD^10, 11^; however, eczema is the cutaneous manifestation of AD and transient wheals and/or angioedema are specific cutaneous manifestations in CSU.

In a previous study, we reported a significant association between epidermal filaggrin expression and urticaria severity. Additionally, the filaggrin breakdown product (FBP), *cis*-urocanic acid (UCA) was found to enhance mast cell degranulation in a mouse model^12^.

Therefore, in the present study, we sought to compare the levels of FBPs, including pyrrolidone carboxylic acid (PCA) and the two isomers of UCA (*cis-* and *trans-*UCA), in skin from patients with CSU and AD in comparison to healthy normal controls (NCs). Furthermore, we attempted to outline the biologic roles of FBPs in the pathogenesis of CSU.

## Results

### TEWL and epidermal pH differ between patients with CSU and AD

Table 1 lists the TEWL amounts and skin pHs of the study subjects. Both TEWL (12.9 ± 4.2) and pH (5.1 ± 0.4) were significantly lower in CSU lesional skin than in the lesional skin from AD patients (29.7 ± 16.3, *P* < 0.001 and 5.5 ± 0.3, *P* = 0.023; respectively).

**Table 1:** Clinical characteristics of the study groups.

	NC (*n* = 10)	CSU (*n* = 10)	AD (*n* = 10)	*P* value	
				CSU vs. AD	CSU vs. NC
**Age (years)** ^*^	39.5 ± 5.3	37.2 ± 10.1	25.3 ± 4.0	0.01	0.631
**Women**	6 (60%)	3 (30%)	3 (30%)	1.00	0.370
**Atopy (%)**	0	9 (90%)	9 (90%)	1.00	<0.001
**Total IgE (KU/L)** ^**‡**^	0.014 (0.008, 0.042)	0.22 (0.096, 0.362)	2.37 (0.45, 5.00)	0.015	0.028
**UAS-Ye (0–15)** ^*^	na	10.8 ± 2.0	na	na	na
**SCORAD** ^*^	na	na	25.8 ± 5.8	na	na
**TEWL (g/h/m** ^**2**^ **)** ^*^					
**Non-lesional**	10.9 ± 2.6	8.6 ± 2.8	11.7 ± 3.6	0.043	0.089
**Lesional**	na	12.9 ± 4.2	29.7 ± 16.3	<0.001	na
**Histamine**	10.9 ± 1.8	13.6 ± 5.7	na	na	0.089
**Epidermal pH** ^*^					
**Non-lesional**	5.5 ± 0.5	4.9 ± 0.4	5.4 ± 0.3	0.017	0.011
**Lesional**	na	5.1 ± 0.4	5.5 ± 0.3	0.023	na
**Histamine**	5.6 ± 0.3	5.2 ± 0.2	na	na	0.123

Histamine, histamine-induced wheals; NC, normal controls; AD, atopic dermatitis; CSU, chronic spontaneous urticaria; UAS, urticaria activity score; SCORAD, scoring atopic dermatitis; TEWL, transepidermal water loss; na, not available. *Values given are the mean ± S.D. ^‡^Values given are the median (25^th^ percentile, 75^th^ percentile). *P* values were obtained by the Mann-Whitney *u* test (continuous variables) and Fisher’s exact test (categorical variables).

Non-lesional skin from the CSU group had a lower TEWL and skin pH than non-lesional skin from the AD group (TEWL: 8.6 ± 2.8 vs. 11.7 ± 3.6, *P* = 0.043; pH: 4.9 ± 0.4 vs. 5.4 ± 0.3, *P* = 0.017; respectively), and had lower skin pH, compared to the NC skin (5.5 ± 0.5, *P* = 0.011). However, there was no difference in TEWL between non-lesional skin of CSU patients and NC skin.

### Total FBPs in CSU skin are equal to those in NC skin, but higher than those in AD skin

**Figure 1:**
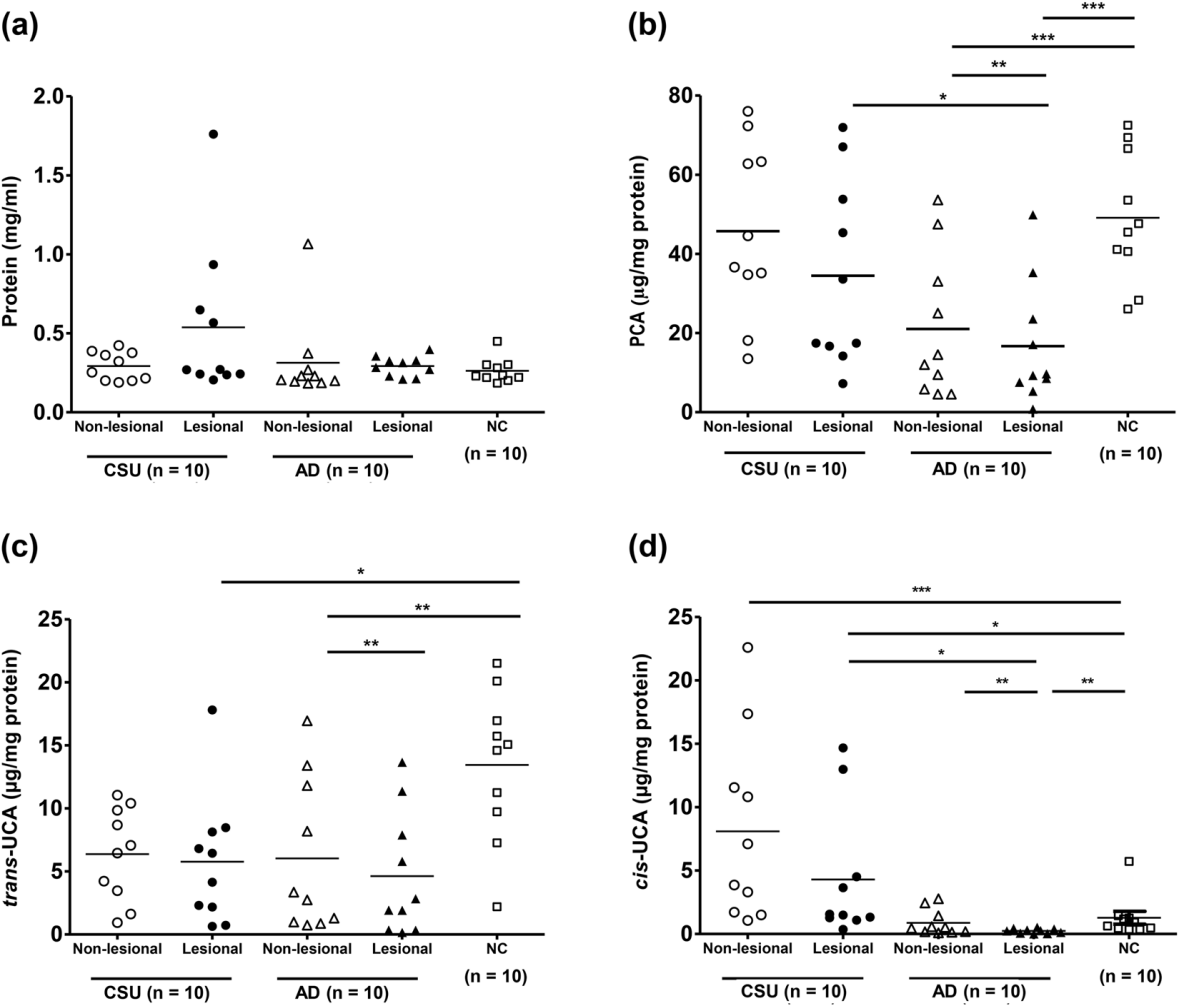
Concentrations of total protein (**a**) and filaggrin break down products, including PCA (**b**), *trans-*UCA (**c**), and *cis-*UCA (**d**), normalised to the quantity of protein from tape-stripped epidermal samples from study subjects. Horizontal lines in the middle of the dot plots indicate mean values. AD, atopic dermatitis; CSU, chronic spontaneous urticaria; Histamine, histamine-induced wheals; NC, normal control. ^*, **, ***^ *P* < 0.05, 0.01, 0.001, obtained by one-way ANOVA with Bonferroni’s post-hoc test.

We compared the quantities of FBPs in SC specimens, including PCA, *cis-*UCA, and *trans-*UCA, among the three study groups (Fig. 1). The total quantities of protein in lesional skins from the CSU and AD groups (0.54 ± 0.49 mg/mL and 0.29 ± 0.06 mg/mL) were not different from the NC group (0.26 ± 0.08 mg/mL) (Fig. 1a). The absolute concentrations of *trans*-UCA (Fig. S1, Supplementary document) in both lesional and non-lesional skin from CSU (*P* < 0.05) and AD subjects (*P* < 0.01) were significantly decreased compared to NC skin. The absolute concentrations of *cis*-UCA in CSU skin were significantly higher than those in AD (*P* < 0.001) and NC skin (*P* < 0.05).

After normalising for total protein content, PCA quantities (non-lesional: 45.72 ± 6.95, lesional: 34.48 ± 7.46 μg/mg protein) levels in CSU skin did not differ from those in NC skin (49.14 ± 5.16 μg/mg protein); however, PCA quantities in both were significantly higher than those found in AD skin (non-lesional: 21.02 ± 5.73 μg/mg protein, lesional: 16.70 ± 4.87 μg/mg protein; *P* < 0.05 for both). The quantity of *trans-*UCA (Fig. 1c) from NC skin specimens (13.44 ± 1.73 μg/mg protein) was significantly greater than those in CSU (non-lesional: 6.38 ± 1.16 μg/mg protein, lesional: 5.76 ± 1.63 μg/mg protein; *P* < 0.01 for both) and AD (non-lesional: 6.03 ± 1.92 μg/mg protein, *P* < 0.05; lesional: 4.62 ± 1.54 μg/mg protein, *P* < 0.01) skin specimens; there were no significant differences in *trans*-UCA level between the CSU and AD groups. The quantities of *cis-*UCA (Fig. 1d) in CSU skin (non-lesional: 8.09 ± 2.34 μg/mg protein, lesional: 4.29 ± 1.64 μg/mg protein) were significantly greater than those in AD (non-lesional: 0.88 ± 0.32, lesional: 0.24 ± 0.06 μg/mg protein; *P* < 0.001 for both) and NC (1.28 ± 0.51 μg/mg protein; *P* < 0.001 for lesional and *P* < 0.05 for non-lesional) skin.

### Ratio of *cis-* to *trans-*UCA is increased in CSU skin

**Figure 2:**
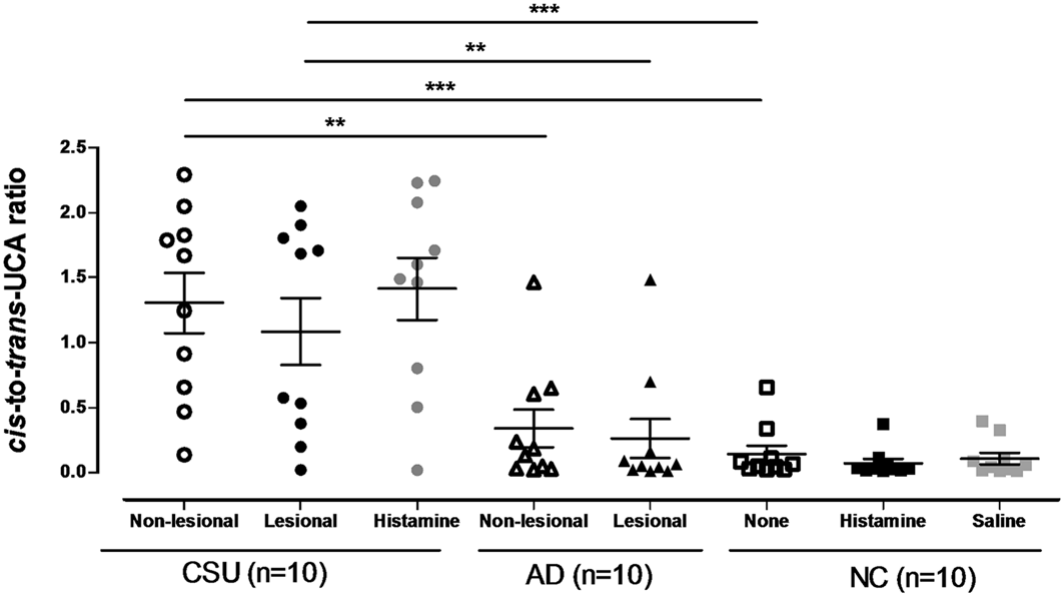
Ratio of *cis-* and *trans-*UCA measured in tape-stripped epidermal samples obtained from study subjects. Horizontal lines in the middle of the dot plots indicate mean values. AD, atopic dermatitis; CSU, chronic spontaneous urticaria; Histamine, histamine-induced wheals; NC, normal control. ^*, **, ***^ *P* < 0.05, 0.01, 0.001, obtained by one-way ANOVA with Bonferroni’s post-hoc test.

The differences in *trans-* and *cis*-UCA concentrations in SC specimens among the three study groups prompted us to investigate the potential use of *cis-*to-*trans-*UCA ratio in differentiating CSU from AD and NC subjects (Fig. 2). The *cis-*to-*trans-*UCA ratio in the SC specimens from CSU patients (non-lesional: 1.30 ± 0.69, lesional: 1.08 ± 0.76) was significantly higher than those in the specimens from AD patients (non-lesional: 0.34 ± 0.43, lesional: 0.26 ± 0.45, *P* < 0.01 for both) and NCs (0.14 ± 0.19; *P* < 0.001 for both non-lesional and lesional). However, no significant difference in *cis*-to-*trans-*UCA ratio was noted between the AD and NC groups.

**Figure 3:**
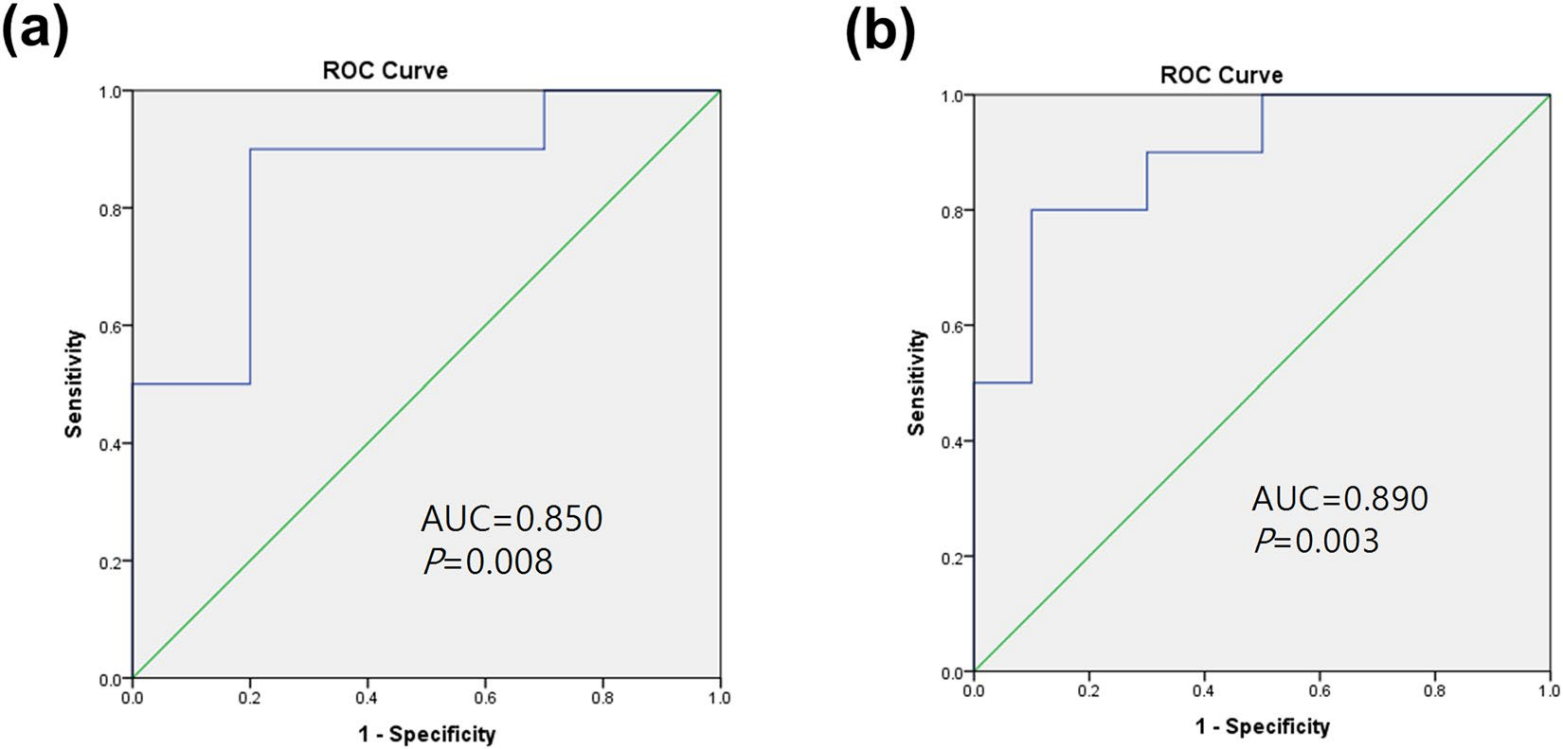
Diagnostic values of *cis-* to *trans-* UCA ratio in distinguishing CSU from AD patients, using receiver operating characteristic curve. Diagnostic values of cis-to trans-UCA ratio in (**a**) lesional skins and (**b**) non-lesional skins are shown. AUC, area under the curve.

Figure 3 shows the diagnostic values of *cis-* to *trans-* UCA ratio in distinguishing CSU from AD patients, using receiver operating characteristic curve. The area under the curve (AUC) for *cis-*to *trans-*UCA ratio in lesional skins was 0.850 (*P* = 0.008) with a sensitivity of 90% and specificity of 80% at the cut-off value of 0.179. In non-lesional skin, the AUC was 0.89 (P = 0.003) with a sensitivity of 80% and specificity of 90% at the cut-off value of 0.653.

### FBPs may contribute to differences in the physiological functions of CSU and AD skin

To investigate whether FBPs in the skin influence the physiological function of the epidermis or disease activity, we assessed associations between FBPs, skin pH, TEWL, and the disease-activity scores of CSU and AD patients. Significantly negative correlations between FBP quantities and TEWL were observed in both lesional (Spearman’s rho = −0.553, *P* = 0.011) and non-lesional (Spearman’s rho = −0.739, *P* < 0.001) SC specimens for all CSU and AD patients. However, no significant correlation was noted between FBPs and pH in either lesional or non-lesional SC specimens from CSU and AD patients.

In the CSU group, there was no significant correlation of FBP quantities with pH or UAS-Ye; however, the pH of lesional skin was positively correlated with UAS-Ye (Spearman’ rho = 0.859, *P* = 0.001). Meanwhile, in the AD group, the quantities of PCA and *trans*-UCA in lesional skin were negatively correlated with both pH (Spearman’ rho = −0.648, *P* = 0.043 and r = −0.729, *P* = 0.017; respectively) and TEWL (Spearman’ rho = −0.650, *P* = 0.042 and r = −0.709, *P* = 0.022; respectively). In addition, the *cis*-to-*trans*-UCA ratio in AD lesional skin was significantly correlated with SCORAD scores (Spearman’ rho = 0.644, *P* = 0.044).

### *Cis-*UCA, but not *trans-*UCA, enhances basophil activation upon IgE stimulation

**Figure 4:**
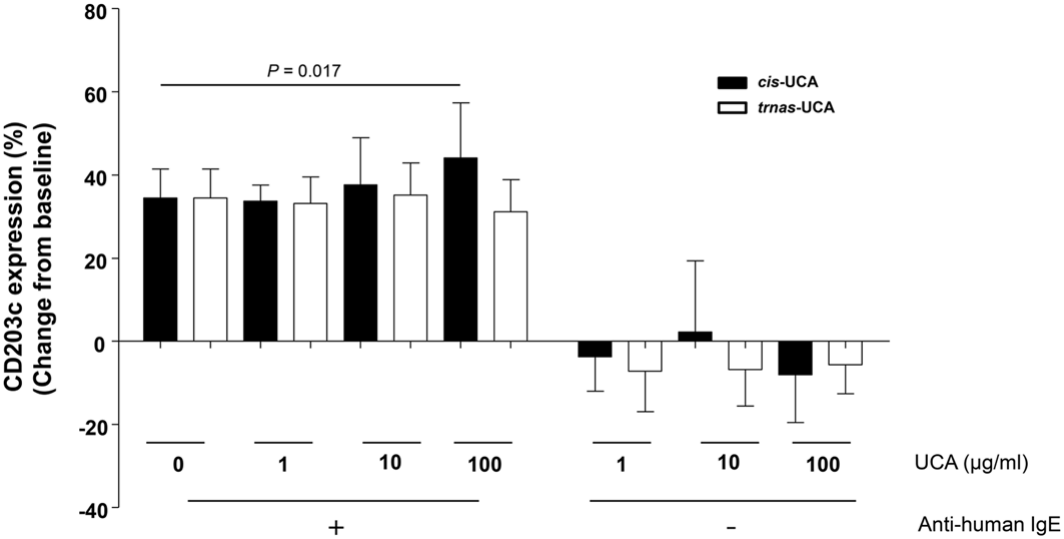
Effects of *cis-* and *trans-*UCA on peripheral blood basophil activation. Basophils from eight atopic subjects were treated simultaneously with *cis-* and *trans-*UCA and with anti-IgE stimulation. Basophil activation was measured by surface CD203c expression using flow cytometry. Changes in the percentages of CD203c expressing basophils from baseline (34.5 ± 7%), where neither anti-human IgE nor UCA was treated, are presented. *P* values were obtained by the Wilcoxon signed-ranks test.

To investigate whether the two UCA isomers have different biological influences on IgE-mediated basophil activation, we performed basophil activation tests with basophils from eight atopic CSU patients. As previously described in the literature and in our preliminary experiments, CD203c had a superior sensitivity than CD63 in evaluating basophil activation^13–15^. Consequently, we measured the expression of CD203c on basophils after incubation with *cis-* or *trans-*UCA (Fig. 4). *Cis-* and *trans-*UCA at the tested concentrations did not induce cell death (data not shown). Pre-treatment of human peripheral blood basophils with *cis-*UCA (100 µg/mL) significantly enhanced the expression of CD203c upon stimulation with IgE (*P* = 0.017), which was not observed in basophils pre-treated with *trans-*UCA. Without IgE stimulation, neither *cis-* or *trans-*UCA affected CD203c expression on basophils.

### *Cis*-UCA enhances degranulation of LAD-2 cells via both IgE-mediated and calcium-mediated pathways

**Figure 5:**
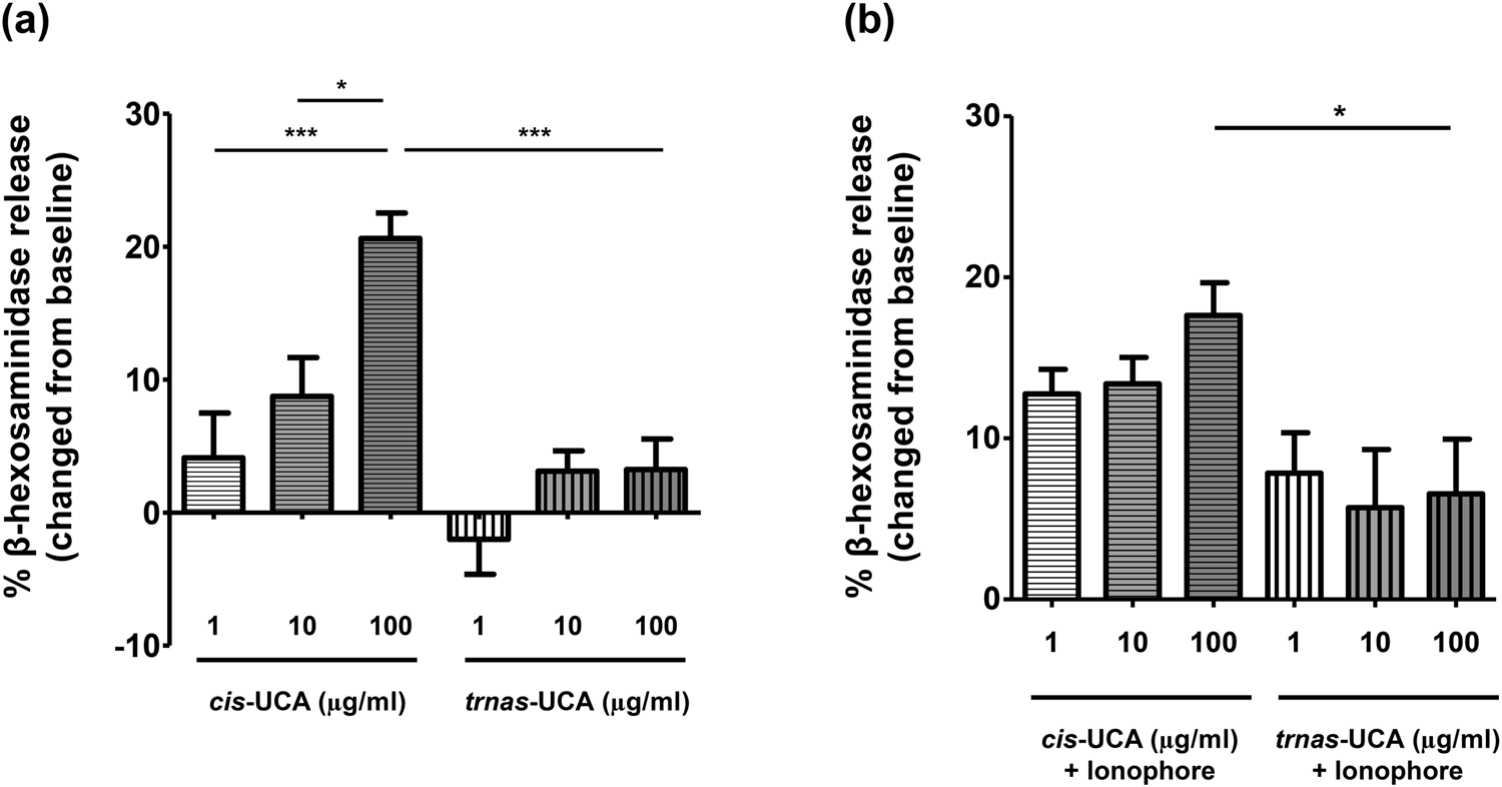
Effect of *cis*- and *trans*-UCA on IgE-mediated (**a**) and calcium-mediated (**b**) degranulation of LAD-2 cells. Mast cell degranulation levels were investigated using the beta-hexosaminidase release test. The control comprised LAD-2 cells sensitised with IgE and stimulated with streptavidin peroxidase (**a**) or only stimulated with calcium ionophore (**b**) without UCA pre-treatment, which yielded 57.5 ± 6.9% and 68.9 ± 9.6% of beta-hexosaminidase release; respectively. *^,^ ***P < 0.05, 0.001, obtained by one-way ANOVA with Bonferroni’s post-hoc test.

To investigate the effect of UCA on LAD-2 cell degranulation via the IgE-mediated pathway, the cells were treated with *cis-* or *trans-*UCA simultaneously with IgE sensitisation for 16 h. Upon stimulation, the IgE-primed LAD-2 cells treated with *cis-*UCA (100 µg/mL) released a significantly higher quantity of beta-hexosaminidase, compared with untreated cells and cells treated with an equal concentration of *trans-*UCA (both *P* < 0.001, Fig. 5a and Fig. S2a, Supplementary document). Consistently, *cis-*UCA enhanced beta-hexosaminidase release from IgE-unprimed LAD-2 cells stimulated with calcium ionophore; moreover, it did so in a dose-dependent manner, which was not observed in *trans-*UCA treated cells (Fig. 5b and Fig. S2b, Supplementary document). Neither *cis-* nor *trans*-UCA affected the expression of FcεRI alpha on LAD-2 cells (assessed by flow cytometry, Fig. S3, Supplementary document). Moreover, we failed in detecting the secretion of inflammatory mediators, including IL-6 and TNFα from LAD-2 cells (assessed by ELISA). The tested concentrations of *cis* and *trans-*UCA did not induce cell death (data not shown).

## Discussion

Previously, we demonstrated increased filaggrin expression in lesional CSU skin at both the mRNA (using quantitative PCR) and protein levels (using immunohistochemistry)^8^. To verify our speculation that increased filaggrin expression leads to increased FBP production, we measured the quantities of PCA and *cis-* and *trans*-UCA in tape-stripped skin specimens from patients with CSU and AD, as well as in NCs. The AD patients showed significantly lower quantities of PCA and total UCA in SC specimens, compared to the NCs; however, the quantities of these substances in the CSU patients did not differ from those in the NC group. Notably, *cis-*to-*trans-*UCA ratio was significantly higher in CSU skin than in both AD and NC skin. We also showed that *cis-*UCA, but not *trans-*UCA, significantly enhanced the activation of human basophils and the degranulation of human mast cells.

Filaggrin in the skin is degraded into its amino acid component, histidine, which is then converted to histamine by histidine decarboxylase or to *trans-*UCA by histidase^16, 17^. *Trans*-UCA is converted into *cis-*UCA by photoisomerisation when the skin is exposed to UV light^8, 18^. We hypothesised that increased filaggrin expression in CSU skin could lead to increased production of histidine and glutamine, resulting in increased synthesis of histamine, UCA, and PCA, respectively^8^. However, PCA and total UCA quantities in the skin of CSU patients and NCs were the same. Thus, we speculated that histidine in CSU skin is predominantly converted into histamine due to a high expression of histidine decarboxylase^19, 20^, leaving the expression of PCA and UCA unchanged. Additionally, a lower expression of filaggrin in AD skin^8^ likely explains the lower productions of PCA and UCA therein, compared to NC and CSU skin, as was observed in the present study. This result is linked clinically to a defect in epidermal barrier function in AD skin.

Interestingly, when we analysed the quantity of each UCA isoform normalised to total protein concentrations in SC specimens, *trans-*UCA levels were significantly decreased, whereas *cis-*UCA levels were significantly increased in the CSU group, compared to the NC group. Consistently, the absolute concentrations of *cis*-UCA were significantly higher in both the lesional and non-lesional skin of CSU patients in comparison to NC and AD skin. Moreover, *cis-*to*-trans-*UCA ratios for both lesional and non-lesional CSU skin were significantly higher than those for AD skin. *Trans*-UCA was the more prominent UCA isoform (contributing up to 80–90% of the total UCA concentration) in AD and NC skin, while *cis*-UCA was more abundant in CSU skin, accounting for 51.5% of the total UCA concentration in non-lesional and 44% of the total UCA concentration in lesional SC specimens. Notably, these ratios were not changed in histamine-induced wheals from CSU patients or NCs, suggesting a persistent increase in *cis-*UCA concentrations across CSU skin. In addition, c*is-* to *trans-*UCA ratio provided good diagnostic values with which to distinguish CSU from AD patients upon applying AUCs of 0.85 for ratio values in lesional skin and 0.89 for ratio values in non-lesional skin. The findings indicate that an increased concentration of *cis*-UCA alone and an increase in *cis*-to-*trans*-UCA ratio in SC specimens could be considered as potential diagnostic markers for distinguishing CSU patients from AD and NC subjects, even before lesions develop.

The underlying mechanisms for an increased *cis-*to-*trans-*UCA ratio in CSU skin have not been studied; however, the conversion between *trans-* and *cis-*UCA is known to be affected by the skin environment. In the present study and others, a significantly lower pH in CSU skin than in AD and NC skin was observed^8^. Meanwhile, the photoisomerisation of *trans-* to *cis-*UCA has been shown to be more efficient at a lower skin pH (3 to 5.5) than at a higher skin pH^21^, explaining the increased *cis-*to-*trans-*UCA ratio in CSU skin. Additionally, *cis-*UCA could be reversed to *trans-*UCA by *cis-*UCA isomerase produced by skin microbial flora, whose function may be affected by the skin pH^22^. The acidic environment noted in CSU skin, therefore, could prevent *cis-* to *trans-*UCA isomerisation, thereby leading to an accumulation of *cis-*UCA and an increased *cis-*to-*trans-*UCA ratio in CSU skin. In this context, we speculate that increased *cis*-UCA production in CSU skin may result from 1) an increased conversion of *trans-*UCA to *cis-*UCA and 2) a decreased conversion of *cis-*UCA back to *trans-*UCA, both are resulted from the acidic nature of CSU skin. Further studies investigating the underlying mechanisms of these phenomena are needed.

Additionally, we observed a higher TEWL and pH in the AD skin, compared to CSU and NC skin, which is consistent with previous findings^8, 23^. PCA and UCA are crucial to maintain epidermal hydration and the acidic environment of the skin^16, 24^. Consequently, decreases in PCA and UCA in AD skin result in impaired hydration and acidity, leading to increased TEWL and skin pH^8, 23, 25^. Also, we observed negative correlations for both PCA and *trans*-UCA with TEWL and pH in AD skin. Taken together, differences in the expressions of PCA and UCA in SC specimens from CSU and AD patients could explain the differences in TEWL and skin pH for these two disease phenotypes.

The dramatic increase in *cis*-to-*trans*-UCA ratio in CSU skin prompted us to investigate the role of *cis*-UCA in the pathomechanism of CSU we with a focus on basophil/mast cell activation, which has long been implicated in the pathogenesis of CSU^26, 27^. A previous *in vivo* study reported that *cis-*UCA at 1 µg/ml significantly induced degranulation of skin mast cells^12^. Several potential mechanisms may account for the *in vivo* degranulation effect of *cis*-UCA on mast cells: c*is*-UCA could induce prostaglandin E2 production, leading to prostaglandin E2 receptor-3-dependent mast cell degranulation^28^. Additionally, c*is*-UCA at approximately 1.38 mg/ml induces neuropeptide release from peripheral sensory nerves in the skin, which, in turn, elicits histamine release from mast cells^29^. In the present study, we observed that *cis-*UCA, but not *trans-*UCA, significantly enhanced human basophil activation, as well as beta-hexosaminidase release, from a human mast cell line (LAD-2) and that this was induced by IgE-mediated pathways. However, expression of a high-affinity receptor for IgE (FcεRI) on LAD-2 cells was not affected by either *cis-* or *trans-*UCA. This finding may suggest that *cis-*UCA could enhance mast cell and basophil degranulation in CSU patients regardless the underlying autoimmune mechanisms. Since intracellular calcium is an important signal for IgE-mediated mast cell degranulation, we investigated whether *cis-*UCA enhances LAD-2 cell degranulation mediated by the calcium signalling pathway. Interestingly, *cis*-UCA also boosted the degranulation of LAD-2 cells induced by calcium ionophore, a reagent that can increase intracellular Ca^2+^ concentrations by facilitating the transport of Ca^2+^ across the plasma membrane. Notably, in the absence of IgE and calcium ionophore, *cis-*UCA could not induce basophil activation or LAD-2 cell degranulation. Accordingly, these findings suggest that *cis*-UCA may enhance IgE-mediated activation of basophils and mast cells by facilitating calcium signalling. A previous study reported that *cis-*UCA could bind to and activate 5-hydroxytryptamine (5-HT) receptor, thereby inducing intracellular calcium mobilisation^30^. Additionally, human mast cells were found to express a variety of 5-HT receptors, and the activation of 5-HT receptors by 5-HT could increase mast cell migration and adherence, although degranulation was not observed^31^.

Studies suggest that anti-IgE monoclonal antibody downregulates the IgE-FcεRI-mast cell axis and may increase the threshold for mast cell activation since omalizumab was introduced for the treatment of CU^32–36^. Our present study demonstrates that *cis*-UCA was able to enhance histamine release from IgE-primed mast cells, but not from unprimed cells. We suggest that *cis*-UCA may be one of the autoreactive components or the final trigger that induces mast cell degranulation upon IgE stimulation. Accordingly, an increase in the epidermal *cis*-to-*trans* UCA ratio in patients whose mast cells and basophils are already primed by IgE may result in increased histamine release. Moreover, neutralisation of circulating IgE by omalizumab treatment may be able to diminish *cis-*UCA enhanced mast cell degranulation. Furthermore, an association between AD and a deficiency in skin FBP, which may lead to increased TEWL and skin pH, was found in our study. Although the *cis*-to-*trans* UCA was decreased in AD versus CSU skin, it correlated significantly with the disease activity score of patients with AD. Taken together, our findings are the first to demonstrate that an altered proportion of FBPs, particularly increased *cis-*UCA concentration, can enhance the degranulation of mast cells and basophils.

Several limitations were raised in the present study. Although the study subjects were enrolled within 3 months to avoid the influence of sunlight exposure, 10 participants in each group would not be sufficient to represent for the inter-individual variations of UCA concentrations in human skins. Filaggrin genetic mutations have been suggested as predisposing factors for approximately 25–50% of AD patients in previous studies^9, 23^; however, we were limited in analysing the associations of genetic polymorphisms of filaggrin in the present study. Additionally, we did not evaluate the *cis*-UCA content in the dermis where cutaneous mast cells exist; however, cis-UCA was found to diffuse freely to the deeper layer of the epidermis with a diffusion coefficient of 10^−7^ m^2^/sec^37^. We could not exclude the possibility of that increased mast cell degranulation leads to the elevated *cis*-UCA in the skin. A further study may be needed to address this issue by comparing the expression of *cis-* and *trans-*UCA in CSU skin before and after an appropriate treatment and between control and uncontrolled CSU groups.

In summary, the present study revealed novel findings related to the CSU pathomechanism, wherein *cis*-UCA may be crucial to increasing skin mast cell degranulation. In particular, we found that *cis*-to-*trans* UCA ratio in the epidermis, even in non-lesional skin, may be potential diagnostic markers to differentiate CSU from AD. We also report an association between AD and a deficiency in skin FBPs, which may lead to increased TEWL and skin pH in AD patients.

## Methods

### Study subjects

Koreans, aged 20–70 years, recruited at Ajou University Hospital, Suwon, South Korea, were enrolled in this study from March to May 2014. We evaluated urticaria activity score (UAS-Ye) modified from the UAS recommended by EAACI /GA2LEN/WAO^38^, which assesses the features of wheals (including quantity [0, no wheals; 1, <10 wheals; 2, 10–50 wheals; 3, >50 wheals], distribution [0, none; 1, <25% of the body surface area (BSA); 2, 25–50% of the BSA; 3, >50% of the BSA], mean diameter [0, no wheals; 1, <1 cm; 2, 1–3 cm; 3, >3 cm], and duration [0, no wheals; 1, <4 hr; 2, 4–12 hr; 3, >12 hr], and intensity of pruritus [0, no pruritus; 1, mild; 2, moderate; 3, severe]) within the last week for outpatient clinic visits, yielding a total score of 0–15. AD was defined according to typical clinical features compatible with the diagnostic criteria for AD suggested by Hanifin and Rajka^39^. The disease activity of AD was evaluated using the SCORing Atopic Dermatitis (SCORAD) as previously described^40^. Patients with CSU did not concomitantly had AD and *vice versa*.

Table 1 shows the clinical characteristics of the three study groups: 10 NCs (mean age, 39.5 ± 5.3 years) with no history of allergy and skin diseases, 10 patients with active CSU (mean age, 37.2 ± 10.1 years; mean urticaria activity score [UAS-Ye] 10.8 ± 2.0), and 10 patients with active AD (mean age, 25.3 ± 4.0 years; mean SCORAD, 25.8 ± 5.8). Atopy was defined by at least one positive skin prick test to common inhalant allergens^41^. Levels of total IgE in blood were measured using the ImmunoCAP system (Thermo-Fisher, Uppsala, Sweden) according to the manufacturer’s instructions. None of the subjects had previously received immunomodulators, including cyclosporine, methotrexate, and anti-IgE. Prior to enrolment, patients had stopped using topical and systemic corticosteroids, as well as topical calcineurin inhibitors, for at least 2 weeks; anti-histamine use was stopped for at least 5 days. None of the healthy controls used anti-histamines. The study was approved by the Institutional Review Board at the Ajou University Medical Center (AJIRB-MED-SMP-13-384, AJIRB-MED-OBS-14-358). All subjects provided written informed consent before participation and methods in this study were performed in accordance with the relevant guidelines and regulations.

To investigate the physiological function of the epidermis, transepidermal water loss (TEWL, Tewameter TM300) and skin surface pH (PH900) on the lesional skin of CSU and AD patients, as well as skin from NCs, were measured.

### Sample preparation

Specimens of the stratum corneum (SC) were obtained using the tape-stripping method with D-squame standard tape (Cuderm, Dallas, TX, diameter, 2.2 cm). Six consecutive tape-stripped specimens were collected from the volar forearm of each subject. For patients with CSU, we collected specimens from both lesional and non-lesional skin on the volar forearm, as well as on histamine-induced wheals. Tape-stripped specimens were collected from both lesional and non-lesional AD skin, as well as from normal skin and histamine-induced wheals of NCs. The specimens were stored at −80 °C.

### Measurement of protein and FBPs in tape-stripping samples

For protein quantification, six tape-stripped specimens were placed in glass vials containing 5 mL of 0.1% (w/v) sodium dodecyl sulphate/2% (w/v) propylene glycol in phosphate buffered saline (PBS) solution, and sonicated for 1 h to obtain soluble proteins. The solutions were then centrifuged at 12,000 rpm for 10 min at 4 °C. After collecting the supernatant, aliquots of the solution were used to quantify protein concentrations; soluble protein concentrations were assayed using a protein assay kit (Pierce, Rockford, IL). The sample plates were incubated for 30 min at 37 °C, after which absorbance was measured with a microplate reader (Spectramax190, MDA) at 595 nm.

With purified standards, FBPs, including PCA and *cis*- and *trans*-UCA, were quantitated using hydrophilic interaction liquid chromatography coupled with tandem mass spectrometry (HILC-MS/MS) for simultaneous determination, according to methods described previously^42^.

### Basophil activation test with UCA

We recruited eight CSU patients who were sensitized with at least one common aeroallergen identified by a skin prick test (Bencard). Venous peripheral blood (10 mL) from each subject was collected into acid-citrate-dextrose containing tubes (BD Vacutainer, Franklin Lakes, NJ, USA), and analysed within 2 hr after collection. Red blood cells (RBC) were lysed in RBC lysis buffer (155 mM NH_4_Cl, 1 mM NaHCO_3_, 0.1 mM EDTA) for 10 min on ice, followed by washing with PBS. Cells were then resuspended in assay buffer (PBS containing 5% bovine serum albumin [BSA, Amresco, Solon, Ohio, USA]), and treated with *cis-* or *trans-*UCA (Sigma-Aldrich, St. Louis, MO, USA) dissolved in PBS, with or without stimulation with 1 μg/mL of goat-anti human IgE (KPL, Gaithersburg, MD, USA) for 30 min at 37 °C. Cells were washed once in PBS, resuspended in assay buffer, and incubated with PE-conjugated anti-human CD203c antibody (basophil activation surface marker), APC-conjugated anti-HLA-DR antibody, and FITC-conjugated anti-human CD123 antibody (BD Biosciences, San Jose, CA, USA) for 20 min at room temperature. Cells were washed once with PBS and analysed by flow cytometry using FACSDiva software, v6.0 (FACSCanto II, BD Biosciences). Basophils were identified as CD123^+^ HLA-DR^−^ populations. The percentage of cells expressing CD203c was evaluated.

### Mast cell degranulation induced by UCA

Laboratory of Allergic Disease 2 (LAD-2) mast cells were kindly provided by Dr. Arnold Kirshenbaum (National Institute of Allergy and Infectious Diseases, Bethesda, MD, USA). Cells were maintained in StemPro-34 medium (Life Technologies, Grand Island, NY, USA) supplemented with 2 mM L-glutamine, 100 U/mL penicillin, 50 μg/mL streptomycin, and 100 ng/mL recombinant human stem cell factor (R&D Systems, Minneapolis, MN, USA) as described previously^43^.

LAD-2 cell degranulation was evaluated by the β-hexosaminidase release test^44^. Cells were treated for 16 h with *cis-* or *trans-*UCA with or without simultaneous sensitisation with biotinylated-IgE (100 ng/mL, BioPorto Diagnostics, Hellerup, Denmark) (IgE-mediated and non-IgE mediated degranulation), followed by 30 min of stimulation with streptavidin peroxidase (100 ng/mL) or calcium ionophore A23187 (1 µM, Sigma) in Tyrode’s buffer containing 0.1% BSA. Total β-hexosaminidase was obtained by lysing LAD-2 cells in 0.1% Triton X-100 in PBS. The supernatants were collected and incubated with an equal volume of p-nitrophenyl N-acetyl-β-D-glucosamide (Sigma, 4 mM in citrate buffer) for 1 h. The reactions were stopped by adding 0.4 M glycine buffer, and the signals were read at 405 nM. The percentage of degranulation was calculated as 100 × (OD stimulated − OD unstimulated)/(OD total lysate − OD unstimulated).

### Statistical analysis

Descriptive statistics are presented for all subjects included in the analysis. Categorical data are presented as numbers and percentages. Continuous data are presented as arithmetic means ± standard deviations or as medians (25^th^ percentile, 75^th^ percentile) when the distribution of the data was skewed. The diagnostic value of *cis*- to *trans-*UCA ratio to distinguish CSU from AD patients was evaluated by using receiver operating characteristic (ROC) curve.

Statistical analyses were conducted using Graph Pad Prism (ver. 4.03; San Diego, CA) and SPSS (ver. 19). Statistical differences between groups were determined using an unpaired t-test or Mann Whitney *u* test, and significance was defined as *P* < 0.05. In comparisons of the two patient groups with the control group, data were analysed by one-way ANOVA, and significant differences in multiple comparisons were determined with Bonferroni correction.

## Electronic supplementary material

Supplementary document
