# Supplementary material for: Increased cis-to-trans urocanic acid ratio in the skin of chronic spontaneous urticaria patients
Source: Sci Rep. 2017 May 2;7:1318. doi: 10.1038/s41598-017-01487-9 (PMC5431012; doi:10.1038/s41598-017-01487-9)
Supplement: Supplementary file 1 — Supplementary document [file 41598_2017_1487_MOESM1_ESM.pdf]

Increased *cis*-to-*trans* urocanic acid ratio

in the skin of chronic spontaneous urticaria patients

Duy Le Pham[1], Kyung-Min Lim[2], Kyung-Mi Joo[3], Hae-Sim Park[1], Donald Y.M. Leung[4], Young-Min Ye[1]

[1]Department of Allergy and Clinical Immunology, Ajou University School of Medicine, Suwon, Republic of Korea;

[2]College of Pharmacy, Ewha Womans University, Seoul, Republic of Korea;

[3]AmorePacific R&D Center, Yongin, Republic of Korea;

[4]Department of Pediatrics, National Jewish Health, Denver, Colorado

Supplementary document

METHODS

Evaluating expression of Fc-epsilon-receptor I (FcεRI) alpha on LAD-2 cells by flow cytometry

LAD-2 cells (1×10^5) were seeded onto each well of a 24-well plate in a complete StemPro-34 medium. LAD-2 cells were treated with *cis*-UCA or *trans*-UCA at different concentrations for 16 hr. Cells were harvested and washed once with PBS. Unspecific binding was blocked by incubating the cells with PBS containing 1% bovine serum albumin for 10 min at room temperature. Cells were incubated with rabbit anti-human FcεRI-alpha IgG antibody (10 μg/ml, Santa Cruz, Dallas, TX, USA) for 20 min on ice, followed by washing with PBS and incubation with an appropriate Alexa Fluor® 488 conjugated secondary body (Invitrogen Life Technologies, Carlsbad, CA, USA) for 20 min on ice. Normal rabbit IgG antibody was used as ab isotype control. Cells were washed, resuspended in PBS and analyzed with a FACS Diva software v6.0 (FACSCanto II, BD Biosciences, Franklin Lakes, NJ, USA).

FIGURE

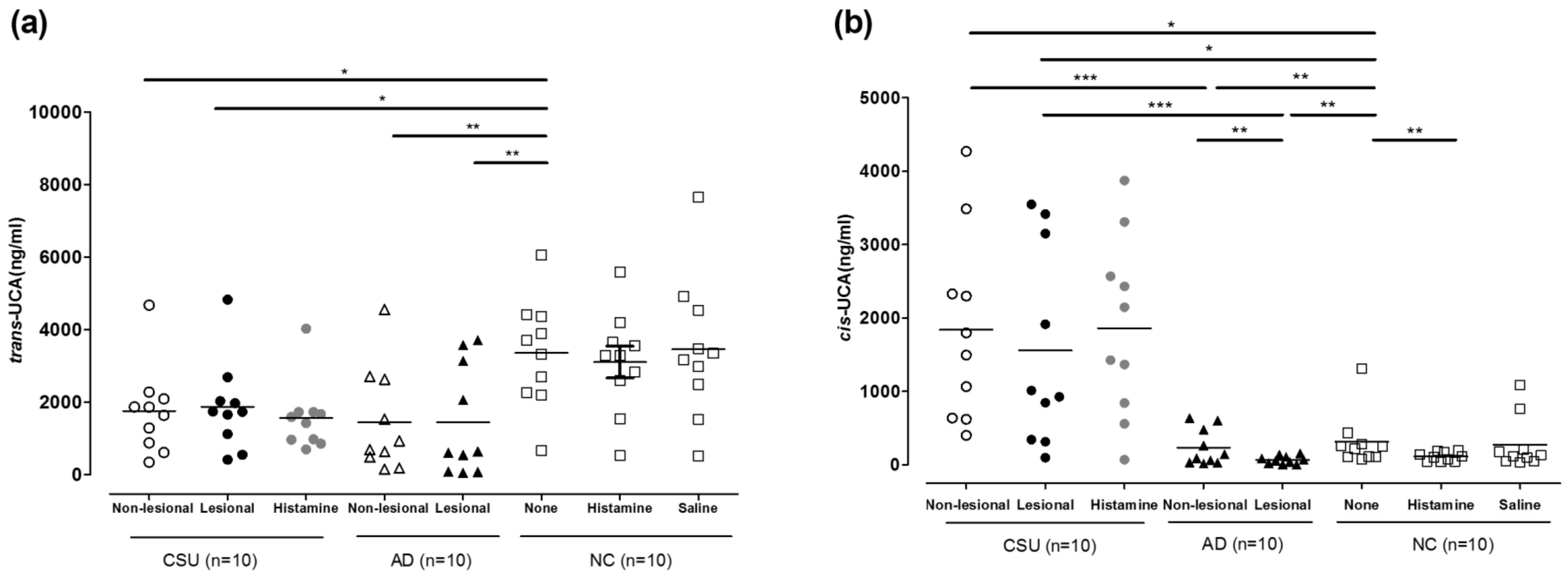

Figure S1. **Absolute concentrations of *trans*-UCA (a) and *cis*-UCA (b) in tape stripped epidermal samples from study subjects**. Horizontal lines in the middle of the dot plots indicate mean values. AD, atopic dermatitis; CSU, chronic spontaneous urticaria; NC, normal control. *, **, ***, $P < 0.05, 0.01, 0.001$, obtained by one-way ANOVA with Bonferroni's post-hoc test.

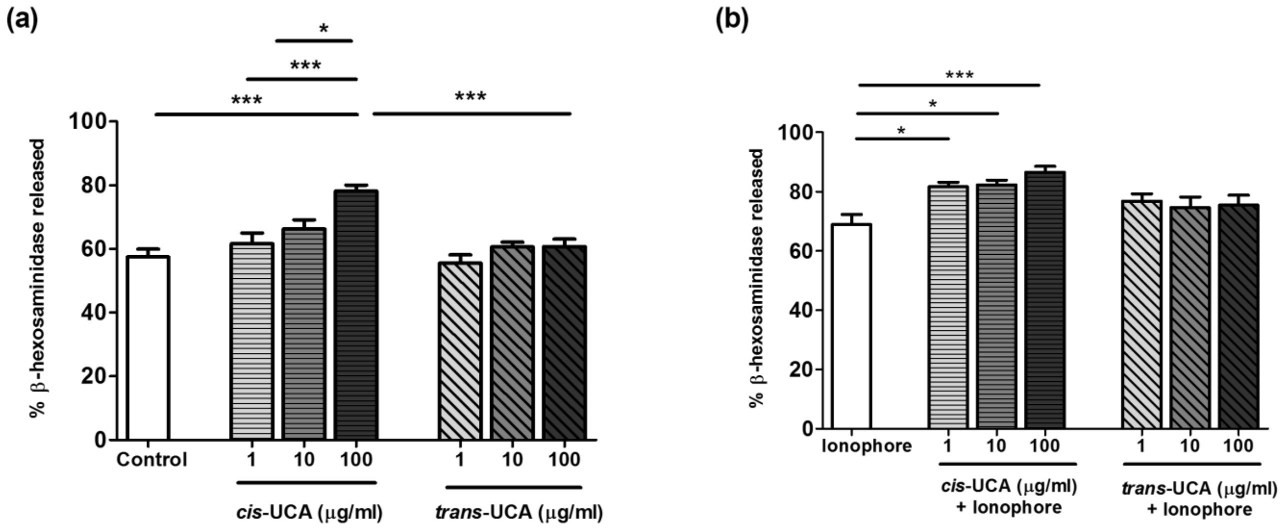

Figure S2. Effect of *cis*- and *trans*-UCA on IgE-mediated (a) and calcium-mediated (b) degranulation of LAD-2 cells. Mast cell degranulation levels were investigated using the beta-hexosaminidase release test. The control comprised LAD-2 cells sensitised with IgE and stimulated with streptavidin peroxidase without UCA pre-treatment. *, *** $P < 0.05$, 0.001, obtained by one-way ANOVA with Bonferroni's post-hoc test.

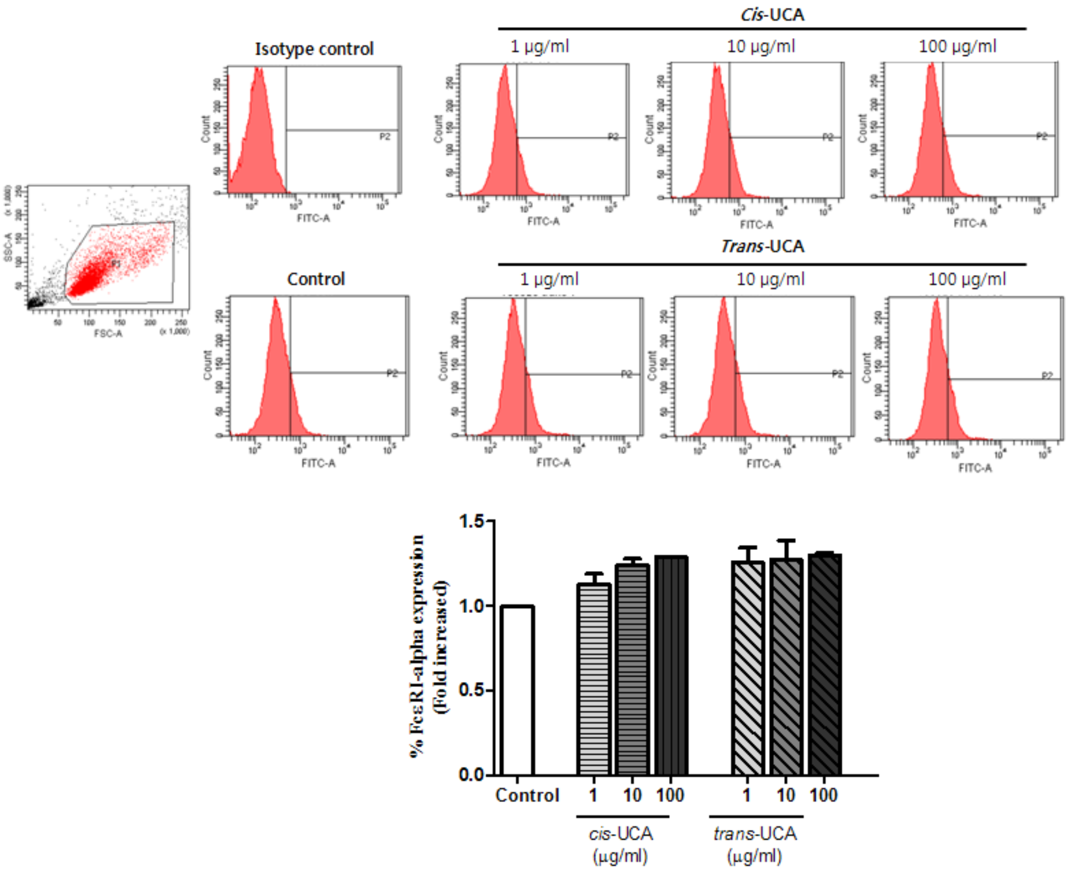

Figure S3. Effect of *cis*- and *trans*-UCA on expression levels of Fc-epsilon-receptor I alpha on LAD-2 cells. Cells were treated *cis*- or *trans*-UCA for 16 hr. Expression of FcεRI-alpha was measured by flow cytometry. No significant differences were observed among the treated groups (analyzed by one-way ANOVA with Bonferroni's post-hoc test).
